# New records of rove-beetles (Insecta, Coleoptera, Staphylinidae) for Azores Islands (Portugal)

**DOI:** 10.3897/BDJ.10.e87672

**Published:** 2022-07-19

**Authors:** Paulo A.V. Borges, Lucas Lamelas-Lopez, Michael Schülke

**Affiliations:** 1 cE3c - Centre for Ecology, Evolution and Environmental Changes, Azorean Biodiversity Group, CHANGE – Global Change and Sustainability Institute, Faculty of Agricultural Sciences and Environment, University of the Azores, PT-9700-042, Angra do Heroísmo, Azores, Portugal cE3c - Centre for Ecology, Evolution and Environmental Changes, Azorean Biodiversity Group, CHANGE – Global Change and Sustainability Institute, Faculty of Agricultural Sciences and Environment, University of the Azores, PT-9700-042 Angra do Heroísmo, Azores Portugal; 2 Blankenfelder Straße 99, D-13127, Berlin, Germany Blankenfelder Straße 99, D-13127 Berlin Germany

**Keywords:** arthropods, Azorean Archipelago habitats, biodiversity, inventory, Staphylinidae

## Abstract

The data we present consist of an updated checklist of the Azorean Staphylinidae (Insecta, Coleoptera), by compiling new identified records of three recent published studies about Azorean arthropods. In general, the records were obtained from different standardised sampling campaigns and from non-standardised observations. The presented records were collected between July 1999 and September 2020, in five islands of the Azores Archipelago: Flores, Graciosa, Terceira, São Miguel and Santa Maria. The samples include records collected in several habitat types, such as native, mixed and exotic forests, pasturelands and agricultural areas (maize fields, orchards, citrus areas and vineyards). This inventory represents the most updated checklist and knowledge about Staphylinidae in Azores and new information includes one new exotic rove-beetle for the Azores (*Thecturotatenuissima* Casey, 1893) and seven new islands records.

## Introduction

The Staphylinidae (Coleoptera) is one of the most diverse families of beetles in Azores and elsewhere. Until 2010, Azorean Staphylinidae knowledge was limited in comparison with other Macaronesian archipelagos (e.g. Assing and Schülke 2006). However, in 2010, the first revised list of Staphylinidae for Azores was published listing 115 species and subspecies (Assing 2010).

More recently, Borges et al. (2022c) published an updated inventory of Staphylinidae of Azores, by collecting more than 10,000 records and identifying a total of 69 species (which represented 51% of the Staphylinidae known for the Archipelago), recording seven new Staphylinidae species for Azores and 66 new island records. In addition, recently two studies were published about Azorean arthropods including Staphylinidae: (1) Borges et al. (2021) provided an inventory of arthropods collected in several Azorean agro-ecosystems, namely citrus orchards, maize fields and vineyards; and (2) Borges et al. (2022e) published and inventory of Azorean arthropods, collected from different habitats types, such as exotic, mixed and native forest patches in four islands of the archipelago.

Given the taxonomic complexity of rove-beetles (Coleoptera, Staphylinidae), it is common that many collected individuals are misidentified or remain identified at genus or family level. In this study, we compiled all these records about Staphylinidae from three previous studies (Borges et al. 2021, Borges et al. 2022c, Borges et al. 2022e), that were posteriorly identified by expert Staphylinidae taxonomists. Consequently, this contribution has the main objective to provide the most updated inventory of Staphylinidae family (Coleoptera, Insecta) for the Azores, by including records collected between July 1999 and September 2020, in several habitat types of five islands of the Archipelago (Flores, Graciosa, Terceira, São Miguel and Santa Maria).

## Study Area and Sampling Description

### Study Area Description

The study area comprise the Azores Archipelago, which is located in the North Atlantic Ocean (37-40°N, 25-31°W), about 1600 km from Europe and 2200 km from North America. The Archipelago, which has volcanic origin, is formed by nine main islands and some small islets. The islands are divided into three main groups: the western group (Corvo and Flores), the central group (Faial, Pico, Graciosa, São Jorge and Terceira) and the eastern group (São Miguel and Santa Maria). The climate is temperate oceanic, with regular and abundant rainfall, high levels of relative humidity, above 95% on average on native forests and persistent winds, mainly during the winter and autumn seasons. The landscape of the islands was dramatically altered as a consequence of human settlement in the 15^th^ century, where native forests were replaced by exotic tree plantations, agricultural and urban areas (Borges et al. 2019). Currently, the native pristine forest represent about 5% of the total surface of the Archipelago, remaining only at the highest and inaccessible areas (Gaspar et al. 2008).

The records of the study were collected on five islands of the Azores Archipelago, Flores, Graciosa, Terceira, S. Miguel and Sta. Maria. The samples were collected in several habitat types, as native, mixed and exotic forests, pasturelands and agricultural areas, as maize fields, orchards, citrus areas and vineyards.

### Sampling Description

This updated checklist of Staphylinidae compile new identified records of three recent published studies about Azorean arthropods (Borges et al. 2021, Borges et al. 2022c, Borges et al. 2022e) by updating the original GBIF datasets of the aforementioned studies (Borges et al. 2022a, Borges et al. 2022b, Borges et al. 2022d). Original data come from different projects, namely: BALA project (see Borges et al. 2016); the Azores LAND-USE project (see Cardoso et al. 2009); AGROECOSERVICES (see Borges et al. 2021); and PRIBES (see Borges et al. 2022e).

In general, the records were obtained from different standardised sampling campaigns and from non-standardised observations. The presented records were collected between July 1999 and September 2020. The sampling methods included Active Aerial Searching, Beating Protocol, Pitfall and SLAM traps. Additional non-standard records are based on cave, colour and malaise traps and direct observations. Specific sampling protocols are detailed on the original publication of each Project (see Borges et al. 2021, Borges et al. 2022c, Borges et al. 2022e). All collected samples were sorted and subsequently identified by an expert taxonomist in the laboratory.

We provide the updated GBIF Event and Occurrence Tables including the new identifications in Suppl. materials 1, 2.

### Taxon Identification and Taconomic Nomenclature

All sorted specimens were identified by a taxonomical expert (MS) using standard methods for the identification of Staphylinidae, that included the extraction of male genitalia when necessary. Specimens were mounted with genitalia also mounted or stored in a small vial and all vouchers are deposited at Entomoteca Dalberto Teixeira Pombo (DTP) at the University of Azores. Taxonomic nomenclature followed Schülke and Smetana (2015).

## New Records

We identified a total of 359 collected Staphylinidae individuals, belonging to 22 species. A total of 18 species are considered introduced (n = 350 individuals) and four native non-endemic (n = 9 individuals).

In this study, we registered a total of eight new records for the islands of Azores (six for Terceira Island and two for São Miguel Island. Four out of the eight records are native non-endemic species. *Thecturotatenuissima* Casey, 1893 is a new record for the Azores Archipelago (Table 1).

*Thecturotatenuissima* Casey, 1893 was sampled in a low elevation maize field in the localitty of São Mateus (Terceira Island). This species is native to the Nearctic, occurring in Canada and United States (Brunke et al. 2021). However, it was introduced to the West Palaearctic, especifically in another Macaronesian archipelagos such as the Canary Islands, where it was recorded as *T.marchii* (Newton 2019, Brunke et al. 2021). The finding of this new exotic species follows the recent tendency to find many new exotic records in Azores (see Borges et al. 2013, Borges et al. 2022e, Borges et al. 2022c).

This publication increases the knowledge on the Azorean beetles and updates the recently-published checklist of Azorean rove-beetles (Borges et al. 2022c). The updated complete checklist of Azorean Staphylinidae is provided in Suppl. material 3, including information about the colonisation status of the species, the island presence, the new records derived from this publication and the synonyms of the species. Now, a total of 137 species and subspecies of rove-beetles (Staphylinidae) are known from the Azores Archipelago. The number of species per island is now as follows: Corvo (4), Flores (65), Faial (66), Pico (62), Graciosa (47), São Jorge (42), Terceira (83), São Miguel (99) and Santa Maria (78). Based on these numbers, it is clear that additional sampling should be performed in Corvo Island.

## Discussion

The Azorean rove-beetle fauna is one of the most diverse within the beetle fauna, but relatively poor in terms of endemic and native species (Borges et al. 2022c). Indeed, with the exception of the recently-described endemic species *Medonvaramontis* Assing, 2013, most of the species recently added for the fauna are exotic species. The recently-published checklist of Azorean Staphylinidae (Borges et al. 2022c; see also Suppl. material 3) added a column in which a new possible tentative categorisation for the colonisation status of the species was created as follows:

- endemic: species for which we have some evidence that they are true endemics, occurring mostly in native habitats.
- doubtfully endemic: species whose status is doubtful, based on our current knowledge on the distribution of congeneric species.
- non-endemic: these include all the previous named as “native” and the since “introduced”, in most cases.
- non-endemic cosmopolitan: these include species with cosmopolitan distribution.

With such categorisation we hope to create a debate on the colonisation status of Azorean beetle fauna and inspire taxonomic revisions that will improve our knowledge on the biogeographical status of the Azorean species.

## Supplementary Material

50A0B1EF-8225-5844-9206-ED87D9D07E9D 10.3897/BDJ.10.e87672.suppl1 Supplementary material 1 Event Table with the new updated data Data type Event Table Brief description Event Table after updating the GBIF datasets from projects PRIBES (Borges et al. 2022a), AZOREAN STAPHYLINIDEA (Borges et al. 2022b) and AGROECOSERVICES (Borges et al. 2022d). File: oo_694465.xlsx https://binary.pensoft.net/file/694465 Paulo A. V. Borges & Lucas Lamelas-Lopez

F50FFB94-63F8-534A-BC96-66274184354E 10.3897/BDJ.10.e87672.suppl2 Supplementary material 2 Occurrence Table with the new updated data Data type Occurrence Table Brief description Occurrence Table after updating the GBIF datasets from projects PRIBES (Borges et al. 2022a), AZOREAN STAPHYLINIDEA (Borges et al. 2022b) and AGROECOSERVICES (Borges et al. 2022d). File: oo_694734.xlsx https://binary.pensoft.net/file/694734 Paulo A. V. Borges

90DCBB5C-AD18-5992-AD74-2E6B7C4CF469 10.3897/BDJ.10.e87672.suppl3 Supplementary material 3 Updated Checklist of Azorean Staphylinidae Data type List of species Brief description Detailed distribution of Azorean Staphylinidae in the nine Azorean islands (AZ - Azores without reference to a given island; COR - Corvo FLO - Flores; FAI - Faial; PIC - Pico; SJG - São Jorge; GRA- Graciosa; TER - Terceira; SMG - São Miguel; SMR - Santa Maria). New records per island are marked. We add also the known taxonomic or nomenclature changes in Azores in four categories (“synonym”, “different combination”, “misidentification” and “emendation/misspelling”).
“1” – Confirmed occurrence, based on Borges et al. (2022c); BALA - records based on BALA protocol (see Borges et al. 2016); LAND-USE (see Cardoso et al. 2009); AGRO – from Borges et al. (2021b); PRIBES (see Borges et al. 2022e) File: oo_694736.xlsx https://binary.pensoft.net/file/694736 Paulo A. V. Borges & Michael Schülke

## Figures and Tables

**Table 1.** Updated inventory of Staphylinidae species collected in five islands of Azores, from 1999 and 2020. The list includes individuals identified at species-level. Scientific name, colonisation status (CS; int = introduced, nat= native non-endemic) and abundance per island are provided. Bold scientific names constitute new records. FLO - Flores; GRA- Graciosa; TER - Terceira; SMG - São Miguel; SMR - Santa Maria.

**Scientific Name**	**CS**	**FLO**	**GRA**	**TER**	**SMG**	**SMR**	**Total**
*Aleocharaclavicornis* L. Redtenbacher, 1849	int	0	0	1	0	0	1
*Amischaforcipata* Mulsant & Rey, 1873	int	0	0	228	0	0	228
*Anotylusnitidulus* (Gravenhorst, 1802)	int	0	0	4	0	0	4
*Athetaatramentaria* (Gyllenhal, 1810)	int	0	0	0	0	1	1
*Athetafungi* (Gravenhorst, 1806)	int	0	0	0	0	7	7
*Athetapasadenae* Bernhauer, 1906	int	1	0	4	0	0	5
*Carpelimuszealandicus* (Sharp, 1900)	int	0	0	1	0	0	1
*Cyphaseminulum* (Erichson, 1839)	int	0	0	9	0	0	9
*Habroceruscapillaricornis* (Gravenhorst, 1806)	int	0	0	3	0	0	3
*Hypomedondebilicornis* (Wollaston, 1857)	int	0	0	8	0	0	8
*Notothectadryochares* (Israelson, 1985)	int	0	0	0	0	42	42
***Oligotapusillima* (Gravenhorst, 1806)**	int	0	0	**1**	0	0	1
***Paraphloeostibagayndahensis* (MacLeay, 1871)**	int	0	0	0	0	**1**	1
***Philonthuslongicornis* Stephens, 1832**	int	0	0	**1**	0	0	1
***Phloeoporacorticalis* (Gravenhorst, 1802)**	nat	0	0	**5**	0	0	5
*Platystethusnitens* (Sahlberg, 1832)	nat	0	0	1	0	0	1
***Pseudomedonobscurellus* (Erichson, 1840)**	nat	0	0	**1**	0	0	1
***Scopaeusportai* Luze, 1910**	nat	0	0	**1**	0	**1**	2
*Stenomastaxmadeirae* Assing, 2003	int	0	1	31	0	0	32
*Suniuspropinquus* (Brisout de Barneville, 1867)	int	0	0	3	0	0	3
***Thecturotatenuissima* Casey, 1893**	int	0	0	**1**	0	0	1
*Trichiusarobustula* Casey, 1893	int	0	0	2	0	0	2
